# Data quantifying interseeded cover crops effects on soil water and corn productivity in corn-soybean-wheat no-till cropping systems

**DOI:** 10.1016/j.dib.2023.109465

**Published:** 2023-08-02

**Authors:** Harry H. Schomberg, Kathryn E. White, Alondra I. Thompson, Steven B. Mirsky

**Affiliations:** United States Department of Agriculture, Agricultural Research Service, Beltsville Agricultural Research Center, Sustainable Agricultural Systems Laboratory, 10300 Baltimore Avenue, Beltsville, MD 20705

**Keywords:** Evapotranspiration, Infiltration, Soil water storage, Water use efficiency, *Secale cereale*, *Vicia villosa*, *Trifolium incarnatum*, *Trifolium pratense*

## Abstract

The data described support the research article entitled “*Interseeded cover crop mixtures influence soil water storage during the corn phase of corn-soybean-wheat no-till cropping systems*”. Data were collected during the corn (*Zea mays* L.) phase from rotations with four different cover crop (CC) treatments. The study was conducted at the USDA research facility in Beltsville, MD from 2017 through 2020. The data are available from a repository at Ag Data Commons. Descriptions of crop rotations, soil water and temperature sensors, placement, and frequency of measurements are provided in the manuscript and repository. Hourly volumetric soil water content (m^3^ m^−3^) (VWC) and soil temperature (°C) data for each soil depth (0-12, 25-35, 50-60, 75-85 cm) are available from the repository. In the manuscript, daily values of soil water storage were used to estimate daily evapotranspiration (ET) and infiltration. A text file of meta information is provided in the repository describing data collection procedures, estimation of ET and infiltration, and methods used to replace sensor data having errors. Daily precipitation, maximum and minimum temperatures, net solar radiation, and windspeed collected at a nearby weather station are provided for estimating growing degree days and potential ET. Cover crop biomass (kg ha^−1^) prior to corn planting and corn yields are provided by replication and cover crop system treatment for the four years.

Specifications Table

**Table:** 

Subject	Agronomy and Crop Science, Soil Science
Specific subject area	Soil water availability, cover crop management, cropping systems management, conservation cropping.
Type of data	Tables Metadata Collected data
How the data were acquired	A nearby weather station provided daily maximum and minimum air temperatures, rainfall, and net solar radiation. Aboveground cover crop biomass was determined from two 0.5 m^2^ areas in each plot prior to corn planting each year. Methods for collection of soil water content and soil temperature are provided in the manuscript [1]. Soil water storage (mm; SWS) was estimated on a daily basis by multiplying VWC by soil layer depth and summing for the profile. Changes in daily SWS were used to estimate daily ET and infiltration [3] and presented as cumulative for the growing season. Data to make these calculations are available from the repository. Corn yield was measured from the whole plot using a combine equipped with a yield monitor.
Data format	Raw Analyzed Filtered
Description of data collection	The data collection includes background information about the experimental treatments, weather data for each year from a nearby weather station, soil water content and soil temperature data on an hourly basis and by depth, calculated values of change in profile soil water content on an hourly and daily basis, estimations of evapotranspiration and infiltration. Available data also include cover crop biomass and corn yields per treatment and replication.
Data source location	**Institution**: United States Department of Agriculture, Agricultural Research Service, Beltsville Agricultural Research Center, Sustainable Agricultural Systems Laboratory **City/Town/Region**: Beltsville, Maryland **Country**: USA **Latitude and longitude (and GPS coordinates, if possible) for collected samples/data:** 39°00’46.7''N 76°56’26.4''W [39.012972, -76.940666] **Field outline:** POLYGON ((-76.941013634205 39.012626386656, -76.942492788658 39.014403170041, -76.941490899771 39.014857604037, -76.940072178841 39.012979640024, -76.937086880207 39.010860093348, -76.937706302851 39.010299683291))
Data accessibility	Repository name: Ag Data Commons, U.S. Department of Agriculture Data identification number: 1d78bd91-5e1a-4d97-b25d-be1dcae4f592 Direct URL to data: https://data.nal.usda.gov/dataset/data-interseeded-cover-crop-mixtures-influence-soil-water-storage-during-corn-phase-corn-soybean-wheat-no-till-cropping-systems-0.
Related research article	H.H. Schomberg, K.E. White, A.I. Thompson, G.A. Bagley, A. Burke, G. Garst, K.A. Bybee-Finley, S.B. Mirsky, Interseeded cover crop mixtures influence soil water storage during the corn phase of corn-soybean-wheat no-till cropping systems, Agricultural Water Management. 278 (2023) 108167. https://doi.org/10.1016/j.agwat.2023.108167.

## 1 Value of the Data

• These data provide information on the effects of presence or absence of CCs on soil water and corn grain yield in the USA mid-Atlantic region. The data are unique because CCs were interseeded into the cash crop grown prior corn.
• These data would be useful to scientists developing models that incorporate CC effects on soil water availability to subsequent crops and to agronomists developing cropping system management strategies that incorporate CCs into rotations.
• These data can be used to calibrate or validate soil water and soil temperature models, model water use by corn in different CC cropping systems and contribute to meta-analyses describing effects of CCs on soil water availability.

## 2 Objective

The research data presented here are from a study undertaken to evaluate the effects of CC management on crop productivity in no-till corn-soybean (*Glycine max* L.)-wheat (*Triticum aestivum* L.) production systems. The study is designated as the Cover Crop Systems Project (CCSP). We focused on four CC treatments immediately prior to the corn cropping phase; two with no CC (NC) prior to corn and two with CC interseeded into the double crop soybean or wheat grown prior to corn. Interseeding CCs can aid their establishment and potential for increased biomass production the following spring. Effects of interseeded CC on soil temperature, soil water balance, evapotranspiration (ET), infiltration, water use efficiency and corn grain yield were evaluated over four years. Data available in the repository [2] adds value to the published paper [1], by providing soil water and soil temperature data from individual depths which are presented in the manuscript as whole profile sums and averages. The greater granularity of the data in the repository allows for evaluation of system responses for each soil layer and would be useful for modelling or meta-analysis.

## 3 Data Description

**Fig 1:**
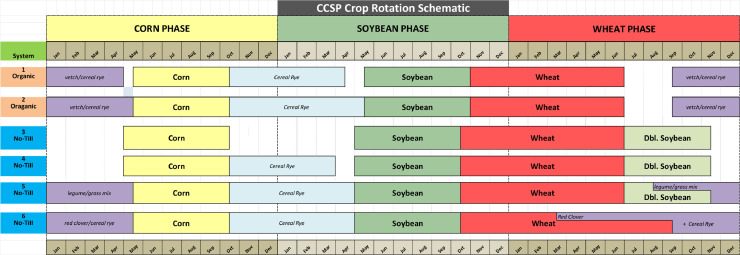
Schematic of the six crop rotations in the Cover Crop Systems Project. The first two systems are organic and the next four are conventional production systems. Only systems 3 through 6 were investigated in this manuscript.

The data files in the repository [2] provide background information and data to support the published manuscript [1]. Within the repository, meta information about the study is included in the Word document file CCSP 2023 AGWAT Metadata.doc. This file includes a brief description of each of the Excel data files included in the repository. The Excel file CCSP Experiment Setup Info Tables 1 Through 4.xlxs provides a schematic of the crop rotation (Fig. 1) and reproduces the first four tables in the manuscript. The four tables contain: (1) a list of the cash crops and CCs making up the four cropping systems; (2) cover crop termination date, corn planting and harvest dates, potential ET, cumulative growing degree days (GDD), rainfall, and sensor measurement periods for each corn growing season; (3) soil water sensor information including types, numbers per plot, waveguide lengths, depths measured, and horizon thicknesses used for estimating water volume; and (4) the average monthly air temperature and rainfall estimated from data collected at the site from 2011 through 2020. Information on the cover crop varieties, seeding rates, month of planting and kill dates are also provided in a worksheet in this file.

Daily weather data used to calculate the 10-yr average monthly air temperature and rainfall during the corn growing season in years 2011 through 2020 are in the file CCSP Weather 2017-2020 Rain and Air Temp forGDD.xlxs. Daily weather data in the same file for the corn growing seasons in 2017 through 2020 were used to calculate corn GDD (°C) using 10 °C and 30 °C as minimum and maximum optimum temperatures as described in Abendroth et al. [4]. Daily weather data used as inputs for ETCalc [5], an online ET calculator, are provided in the file CCSP ETCalcInput OutputData andMetaInfo.xlxs along with the output from the calculator.

Daily values of soil water (mm) and soil temperature (°C) measured during the corn phase for each year are included in the Excel file CCSP Soil Temperature And Soil Water ByDepth.xlxs. Data are provided in separate tabs for each year at the replication, CC treatment, and depth levels. The whole profile soil water data (sum of all depths) used to estimate daily ET and infiltration are included in this file.

Cover crop biomass (kg ha^−1^) prior to corn planting and corn yields (Mg ha^−1^) for the four years are in the Excel file CCSP Corn Yield Cover Crop Biomass.xlsx. Data are provided at the replication and CC treatment level for each year.

## 4 Experimental Design, Materials and Methods

### 4.1 Study site

This field study was conducted at the Beltsville Agricultural Research Center (BARC) in Beltsville, MD USA (39^◦^00′ 51.3''N, 76^◦^56′ 29.0''W) from 2017 to 2020. Details about the soils and previous history of the site are given in the manuscript [1]. The field study uses a randomized split-plot design with four replications (blocks). Each rotation phase crop (corn, soybean, or wheat) is present in each block every year and phases are considered the whole plots. Cash crops are rotated to a different group of plots in each replication each year. For example, corn is grown in group A plots in year 1, in group B in year 2 and in group C in year three. In the fourth year, corn returns to the group A plots.

For this dataset, the experimental design is simplified to a completely randomized block design because soil water data was collected only during the corn phase of the rotation every year. Six cover crop management treatments constitute split-plot treatments. Systems 1 and 2 are in organic management and are not included in this manuscript. Systems 3 and 4 are no-till corn–soybean–wheat–double crop soybean (DCS) rotations and do not have a cover crop (NC) prior to corn. In System 4, a cereal rye CC is grown between corn harvest and planting of soybean while System 3 does not include this cereal rye CC. System 5 is a no-till corn–soybean–wheat–DCS rotation that adds a rye + legume mix [hairy vetch (*Vicia villosa* Roth) + crimson clover (*Trifolium incarnatum* L.)] interseeded into the DCS prior to canopy closure in mid-August. System 6 is a no-till corn–soybean–wheat rotation that includes a red clover CC interseeded into wheat at green up in March. Cereal rye CC is interseeded into the growing red clover after wheat harvest in July.

### 4.2 Agronomics

Details on management of crops and CCs are provided in the manuscript [1] and followed University of Maryland Extension management recommendations. Corn in Systems (3 and 4) was planted in early May, usually 10 to 20 days prior to planting in Systems (5 and 6), except in 2020 when all systems were planted on the same day. The corn variety all years was Pioneer 0506AM. Systems were designed to mimic farmer practices in the Mid-Atlantic states. Some producers do not grow CCs or kill them early near the end of April so as to avoid the CCs interfering with corn planting (Systems 3 and 4). Other growers maximize CC biomass production by allowing the CCs to grow until just prior to corn planting (Systems 5 and 6). Information on the cover crop varieties, seeding rates, month of planting and kill dates are provided in the file CCSP Experiment Setup Info Tables 1 Through 4.xlxs.

Cover crops in Systems 5 and 6 were terminated with herbicides and allowed to remain standing until the corn planting operation which flattened the majority of the residues creating a CC mulch layer on the soil surface. Data for CC aboveground biomass sampled just before termination are provided in the file CCSP Corn Yield Cover Crop Biomass.xlsx.

### 4.3 Soil water and temperature measurements

Soil volumetric water content (m^3^ m^−3^) and soil temperature (°C) were measured in plots in two (2017) or three (2018 – 2020) blocks using time-domain reflectometry (TDR) sensors. Sensors were installed in a vertical orientation thus measuring soil layers of 0-12, 25-35, 50-60, 75-85 cm. Data were collected hourly (2017) or at 15 minute intervals (2018 – 2020) as described in the manuscript [1].

### 4.4 Data QAQC

Raw volumetric water content data were evaluated for quality assurance using routines from the International Soil Moisture Network QAQC Flagit routine (available from Github) [6,7]. Flagged data were evaluated with graphs to compare with data from nearby plots. Details about missing data for each year and solutions taken to replace missing data for each year are provided in the file “CCSP 2023 AGWAT Metadata.docx” in the repository [2]. Missing volumetric water content and soil temperature data due to bad sensors or failure of data loggers as a percentage of the total data collected was 0.13, 0.17, 3.57 and 0.23 % for 2017, 2018, 2019 and 2020, respectively.

### 4.5 Estimation of soil water storage, evapotranspiration and infiltration

**Fig. 2:**
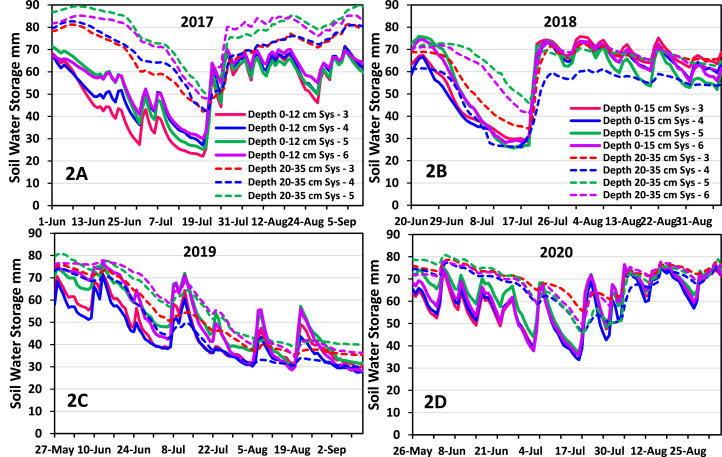
Soil water storage in four cover crop systems at the upper two depths during the corn growing season. Data illustrate changes in soil water storage in 2017 (A), 2018 (B), 2019 (C) and 2020 (D). The upper depth in 2017 was 0- to 12-cm and 0- to 15-cm in the other years. The legend in 2B applies for 2C and 2D.

The data set in the repository contains the hourly and 15-minute volumetric water content and soil temperature data used to calculate daily values used in the manuscript. Data from all sensors within a depth were averaged after data cleaning for each measurement interval. Soil water storage as mm water was calculated for each measurement period by multiplying the volumetric water content by the soil depth interval. In the manuscript [1], millimeters of water for each depth were summed to obtain whole soil profile (0–800 mm) soil water storage and used to estimate evapotranspiration and infiltration [3]. The graphs in Fig. 2A through Fig. 2D provide soil water storage data for the top two depths in each of the CC systems during the four corn growing seasons. The graphs illustrate the response of soil water storage to rainfall and soil drying events for the two depths. Greater granularity of data in the repository will allow for its use in evaluation of system responses for each soil layer and can be useful for modelling CC effects on soil water availability to corn in conservation tillage systems. The data will also be useful for meta-analysis of CC influences on soil water availability.

## Ethics Statements

The work outlined above did not involve human or animal subjects; therefore, no regulatory compliance guidelines were applicable.

The U.S. Department of Agriculture (USDA) prohibits discrimination in all its programs and activities on the basis of race, color, national origin, gender, religion, age, disability, political beliefs, sexual orientation, or marital or family status. USDA is an equal opportunity provider and employer.

## Disclaimer

Trade names are necessary to report factually on available data; however, the USDA neither guarantees nor warrants the standard of the product or service. The use of the name by USDA implies no approval of the product or service to exclude others that may also be suitable. Any use of trade, firm, or product names is for descriptive purposes only and does not imply endorsement by the U.S. Government.

## CRediT authorship contribution statement

**Harry H. Schomberg:** Conceptualization, Formal analysis, Investigation, Resources, Writing – original draft, Visualization, Writing – review & editing. **Kathryn E. White:** Writing – original draft, Writing – review & editing. **Alondra I. Thompson:** Investigation, Data curation, Writing – review & editing, Visualization. **Steven B. Mirsky:** Conceptualization, Writing – review & editing, Resources.

## Data Availability

Data from: Interseeded cover crop mixtures influence soil water storage during the corn phase of corn-soybean-wheat no-till cropping systems (Original data) (USDA Ag Data Commons).
